# Diatom communities in the High Arctic aquatic habitats of northern Spitsbergen (Svalbard)

**DOI:** 10.1007/s00300-016-2014-y

**Published:** 2016-08-04

**Authors:** Aleksandra Zgrundo, Barbara Wojtasik, Peter Convey, Roksana Majewska

**Affiliations:** 1 Faculty of Oceanography and Geography, University of Gdansk, Al. Piłsudskiego 46, 81-378 Gdynia, Poland; 2 Faculty of Biology, University of Gdansk, ul. Wita Stwosza 59, 80-308 Gdańsk, Poland; 3 British Antarctic Survey, Natural Environment Research Council, High Cross, Madingley Road, Cambridge, CB30ET UK; 4 BioNEM Laboratory, Department of Experimental and Clinical Medicine, University “Magna Græcia” of Catanzaro, Loc. Germaneto, 88100 Catanzaro, Italy; 5 School of Biological Sciences, Faculty of Natural Sciences, North-West University, Potchefstroom, 2520 South Africa

**Keywords:** Diatom absence, Diatom assemblages, Frustule dissolution, Lakes, Sediment analysis, Polar

## Abstract

As High Arctic environments are particularly sensitive to global and regional climate changes, a growing number of studies have focused on that region. It has been shown that living and fossil diatoms can be successfully used to track environmental changes in polar habitats. Nevertheless, the diatom flora of many Arctic areas remains unknown. The present study set out to examine the diatom flora in the rarely visited and near-pristine zone of northern Spitsbergen. Examination by light and scanning electron microscopy of 25 sediment samples, collected in fjords, tidal plains and lakes, indicated significant differences between the diatom assemblages identified in lakes located within different fjord watersheds. Altogether, 96 diatom taxa (46 genera) were found. The most abundant species (*Achnanthidium minutissimum*, *Staurosirella pinnata* and *Nitzschia alpina*) occurred in at least eight of the 11 investigated lakes. Assemblages from the Woodfjorden region were characterized by the presence of *Cavinula pseudoscutiformis* and *Encyonema reichardtii*, along with *Navicula* spp., which coincided with relatively low conductivity (34–58.7 µS cm^−1^) and near-neutral pH (7.2–7.5). Diatom assemblages found in the Wijdefjorden area were typically characterized by *Denticula kuetzingii* and *Nitzschia inconspicua*, with these lakes generally having higher water conductivity (>184 µS cm^−1^) and pH (7.5–8.1) conditions. Conductivity, biogenic silica concentration and water temperature were indicated as significant predictors of diatom community species composition and structure. No diatom frustules were found in fjord and tidal plain sediments. The effects of selected environmental factors on diatom assemblage formation are discussed.

## Introduction

At present, some of the most prominent signs of climate change can be observed in the Arctic region (ACIA 2005; Pachauri and Meyer 2014). Recent studies, however, show that the effects of climate change may be different from previously thought and that the cascading effects associated with it will be manifested in different complex ways in different parts of the world (Kriegler et al. 2012). Improved knowledge of the multiple and interacting effects of such changes on both abiotic and biotic elements of various ecosystems will be essential in the prediction of future shifts in global biodiversity and Earth’s biogeochemical cycles (Falkowski et al. 2008; Dawson et al. 2011; Convey et al. 2012). It is therefore necessary to gather detailed information on a variety of habitats that are already or may soon be experiencing such changes. As the urgent need for broadly based programmes of research has increasingly been recognized, studies have started to focus on the Arctic region (e.g. Wassmann et al. 2011; Camill et al. 2012; Convey et al. 2012; Coulson et al. 2014; Roberts et al. 2015). Nevertheless, relatively limited information is available on the ecology of many Arctic aquatic habitats, especially those located in the High Arctic. These habitats offer a unique opportunity to study the multi-factorial impacts of climate change on selected groups of organisms, while studies conducted in remote Arctic regions may be particularly useful and valuable for the assessment and better understanding of the potential consequences of climate change.

Enhanced warming of high-latitude regions relative to the global average (known as “polar amplification”) is a commonly accepted notion, but the limited length of the instrumental data record is a serious obstacle to addressing many crucial questions regarding environmental and climatic changes over the longer term (Smol and Douglas 2007). Therefore, other tools and approaches are applied in investigations of ecosystem change over time. Arctic lake and pond sediments, for instance, are considered natural recorders of temporal variations as they may contain various indicators of past and present physical, chemical and biological conditions (Douglas et al. 1994). Among such indicators, diatoms are among the most commonly used thanks to their high sensitivity and quantifiable optima to numerous environmental variables (Stoermer and Smol 1999; Battarbee et al. 2001; Michelutti et al. 2013). Although they have long and successfully been used in paleoecological and limnological studies worldwide, including in the Arctic region (Koç and Jansen 1992; Koç et al. 1993; Birks and Koç 2002; Bouchard et al. 2004; Jessen et al. 2010; Michelutti et al. 2006, 2013), information on diatoms from Svalbard (High Arctic) aquatic habitats is at best fragmentary (Picińska-Fałtynowicz 1988; Beyens and Van de Vijver 2000; Jones and Birks 2004; Kim et al. 2008; Ki et al. 2009; Pinseel et al. 2015), and little is yet known about their contemporary communities in terms of either taxonomic composition or ecological preferences. The “calibration” of modern diatom assemblages to present-day conditions is necessary to enable inferences to be drawn on past or future environmental changes (Michelutti et al. 2006).

Here, we present the first report on the diatom flora of a remote and relatively rarely visited region of northern Svalbard. The aim of this study was to investigate and describe the diatom assemblages of different aquatic habitats in northern Spitsbergen in relation to their environment. The survey included fjords, tidal plains and lakes that are characterized by different water chemistry and bio-geo-morphological features. This work contributes to the general understanding of diatom ecology and biogeography in the Svalbard region, and the data obtained have implications for paleoecological investigations in this climatically particularly sensitive zone.

## Materials and methods

### Study area and sampling procedures

**Fig. 1:**
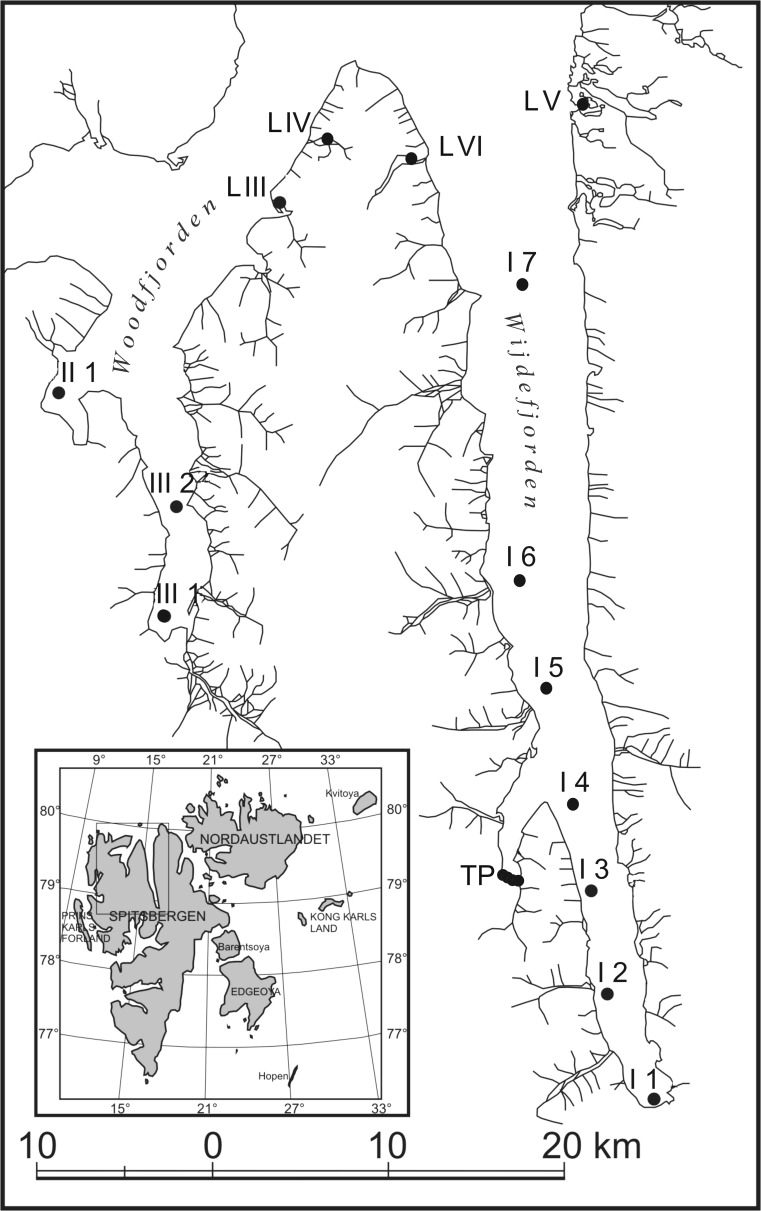
Study area and sampling stations

The study area covered the coastal region of northern Spitsbergen and included the two major fjord systems of Woodfjorden and Wijdefjorden (Fig. 1). Wijdefjorden is the longest (ca. 110 km) of Svalbard’s fjords and reaches a depth of 246 m. Woodfjorden is the fourth longest (64 km) fjord in Svalbard and, together with the smaller Bockfjorden and Liefdefjorden, constitutes a fjord system surrounded by mountains of various geological formations. Its depth is ca. 200 m (Ottesen et al. 2007; Sapota et al. 2009). The region is influenced to varying degrees by both Atlantic and Arctic waters. Generally, Wijdefjorden is more strongly influenced by Arctic water masses, and the impact of Atlantic waters is reported only during periods of high activity of the West Spitsbergen Current. The other fjords are affected by the West Spitsbergen Current for most of the year (Loeng 1991; Walczowski and Piechura 2006; Sapota et al. 2009).

Sampling stations were situated in fjords, tidal plains and lakes and were labelled as I–III for the three fjords (Wijdefjorden, Bockfjorden and Woodfjorden, respectively), IV–VI for the areas near the respective fjord mouths, TP for tidal plains and L for lakes. Arabic numerals were used to identify the stations within selected areas. Only the 25 samples for which the full set of measurements (see *Chemical analyses* section) was obtained are considered in this study. The locations of selected stations together with the most significant environmental data are shown in Table 1. Surface sediment samples were collected with an Ekman grab (23 × 23 cm) between 22 and 30 July 2005 at 34 sampling stations located within the study area (Sapota et al. 2009). The water temperature was measured in situ. Simultaneously, description of sampling sites including, among other factors, their morphological and hydrological characteristics was made (Table 2). For more detailed description of the studied waterbodies, see Sapota et al. (2009).

**Table 1:** Geographical positions of sampling stations and selected environmental characteristics

Location	Site	Date of sampling	Latitude φ N	Longitude λ E	Depth (m)	Temperature (°C)	Conductivity (µS cm^−1^)	Salinity PSU
Fjords	I 1	22.07.2005	78°54.40′	16°24.60′	16	2.7	–	32
	I 2	22.07.2005	79°00.40′	16°19.10′	60	2.1	–	29.3
	I 3	22.07.2005	79°05.00′	16°05.00′	60	3.6	–	29.9
	I 4	22.07.2005	79°10.40′	15°49.30′	56	3.3	–	31.4
	I 5	23.07.2005	79°15.00′	15°44.00′	76	1.3	–	29.9
	I 6	23.07.2005	79°20.10′	15°42.90′	101	2.2	–	34.9
	I 7	24.07.2005	79°38.40′	15°45.30′	16	4.6	–	34.6
	II 1	26.07.2005	79°27.30′	13°22.90′	54	2.8	–	34.8
	III 1	26.07.2005	79°18.80′	13°53.60′	44	1.2	–	30.5
	III 2	26.07.2005	79°23.60′	13°54.00′	75	4.4	–	31.7
Tidal plains	TP I 1	22.07.2005	79°06.08′	15°38.64′	0.3	4.2	–	6.6
	TP I 2	22.07.2005	79°06.05′	15°39.91′	0.3	4.6	–	0.5
	TP I 3	22.07.2005	79°06.06′	15°40.98′	0.3	4.5	–	3.7
	TP I 4	22.07.2005	79°06.05′	15°41.18′	0.3	3.0	–	0
Lakes	L III 1	27.07.2005	79°40.33′	14°15.40′	0.3	7.8	34	0
	L III 2	27.07.2005	79°40.39′	14°15.07′	0.3	8.9	34.1	0
	L III 3	27.07.2005	79°40.90′	14°13.38′	0.3	6.9	35.3	0
	L III 4	27.07.2005	79°40.66′	14°12.15′	0.3	8.6	48.1	0
	L III 5	27.07.2005	79°40.57′	14°12.06′	0.3	9.7	58.7	0
	L III 6	27.07.2005	79°40.53′	14°11.92′	0.3	7.7	1729	0.7
	L IV 1	28.07.2005	79°43.58′	14°24.12′	0.3	6.2	58.2	0
	L V 1	29.07.2005	79°48.18′	15°40.01′	0.3	3.2	28.9	0
	L V 2	29.07.2005	79°48.20′	15°37.87′	0.3	7.1	191.6	0
	L V 3	29.07.2005	79°48.14′	15°37.56′	0.3	7.1	184.4	0
	L VI 1	30.07.2005	79°43.11′	14°53.12′	0.3	6.8	1227	0.4

**Table 2:** Description of the investigated lakes

	LIII1	LIII2	LIII3	LIII4	LIII5	LIII6	LIV1	LV1	LV2	LV3	LVI
Elevation a.s.l. (m)	57	57	6	6	6	1	10	15	20	20	5
Size (ha)	1.2	0.25	0.5	0.5	0.3	0.4	129	812	0.3	0.1	214
Max depth (m)	3.6	0.3	0.5	0.5	0.3	0.3	≥33.8	≥65	0.2	0.1	≥22.6
Distance from the coast (m)	300	300	100	150	150	150	300	800	200	200	200
Vegetation cover	Yes	Yes	Yes	Yes	Yes	No	No	No	No	No	No
Tundra plant cover	Yes	Yes	Yes	Yes	Yes	No	No	No	No	No	No
Inflows	Yes	Yes	No	No	No	No	Yes	Yes	Yes	No	Yes
Incidental sea water inflows	No	No	No	No	No	Yes	No	No	No	No	Yes
Outflow to the sea	Yes	Yes	No	Yes	Yes	No	Yes	Yes	Yes	Yes	Yes
Snow in the catchment	Yes	Yes	No	No	No	No	Yes	Yes	No	No	Yes
Glaciated landscape	No	No	No	No	No	No	Yes	Yes	Yes	No	Yes
Strandflat landscape	No	No	No	No	No	Yes	No	No	No	No	No
Type of surface sediments	Mud	Mud	Sand	Mud	Mud	Muddy sand	Mud	Gravelly sand	Sandy mud	Sand	Gravelly sand
Detritus in sediments	No	No	Yes	No	No	No	No	No	Yes	Yes	Yes

### Chemical analyses

Selected physical and chemical parameters were analysed in the sediment samples obtained: humidity (H), loss on ignition (LOI), total and organic carbon (C_tot_ and C_org_), total nitrogen (N_tot_), biogenic silica (BSi), polychlorinated biphenyls (PCBs), organochlorine pesticides (OCPs) and polycyclic aromatic hydrocarbons (PAHs). Furthermore, in pore waters measurements of nutrients (NH_4_, NO_3_, PO_4_, SiO_4_) were made. The results of a study examining the impact of long-range atmospheric transport of anthropogenic pollutants and their concentrations (PCBs and OCPs) have been published elsewhere (Sapota et al. 2009), and relevant data from that study are drawn on here.

### Diatom identification

Laboratory treatment of sediments for diatom analysis included careful boiling with hydrogen peroxide following the method of Battarbee (1986). Permanent slides were mounted in Naphrax and examined under a Nikon 80i light microscope with differential interference contrast (DIC) at 1000× magnification. On each slide, 300–500 valves were counted on random transects across the slide. For samples where no diatoms were found (tidal plain and fjord samples), a further set of sub-samples was prepared following the method described by Siemińska (1964). In addition, parts of the oxidized suspensions were placed on aluminium stubs and sputter-coated with platinum, and supplementary analysis was performed using a SUPRA 40 (Zeiss) scanning electron microscope (SEM). Diatom identification was based on extensive reviews and comparison with the most up-to-date literature (e.g. Foged 1974, 1981; Krammer and Lange-Bertalot 1986, 1988, 1991a, b; Lange-Bertalot and Metzeltin 1996; Metzeltin and Witkowski 1996; Round and Bukhtiyarova 1996; Johansen and Sray 1998; Lange-Bertalot and Genkal 1999; Lange-Bertalot 2001; Monnier et al. 2007; Antoniades et al. 2008; Mayama and Idei 2009; Alfinito and Lange-Bertalot 2013; Lowe et al. 2014). Samples, slides and SEM stubs are stored at the University of Gdansk (Gdansk, Poland) and are available for further examination.

### Statistical analyses

Statistical analyses were performed using Primer version 6 (Clarke and Gorley 2006) and Canoco version 5.0 (ter Braak and Šmilauer 2012). To evaluate the relationship between the diatom assemblages and measured environmental variables, a constrained ordination method was used. Prior to this analysis, an unconstrained unimodal ordination (detrended correspondence analysis, DCA) was performed and the length of its ordination axes was measured. On this basis (the longest axis = 3.12 turnover units), the unimodal method was selected as the most appropriate for the study dataset (Šmilauer and Lepš 2014). Subsequently, canonical correspondence analysis (CCA) was performed on log-transformed relative abundance data (species relative abundance = the per cent composition of that species to the total number of diatoms found in the sample). As the data were characterized by a high degree of variation and skewed distributions, log transformation was preferred to square-root transformation. All taxa were included in the analysis. A Monte Carlo permutation test was used to test the significance of the axes (4999 permutations, *p* < 0.05). In order to select the best subset of the chosen environmental variables to summarize the variation in diatom composition, interactive forward selection was performed. Uninformative variables were removed by semiautomated stepwise procedure (Draper and Smith 1981) conducted using the CANOCO package. Environmental variables were added one at a time, and CCA was performed on each variable. Subsequently, variables were chosen based on the goodness of fit and a randomization test was used to assess the significance of the correlation found (*p* values). Each time, a partial CCA was performed on the remaining variables separately and the procedure was repeated. The analysis was stopped when the significance level calculated for remaining variables exceeded 0.05. Finally, CCA was performed on all chosen variables together. Categorical (descriptive) variables (Table 2), which provide additional background information for the study, were not included in this analysis. The Bray-Curtis similarity index, calculated using taxa relative abundance data, was used to produce a matrix for non-metric multidimensional scaling (nMDS) ordination, and a plot was created to visualize the differences/similarities among samples. To test for significant differences between the diatom assemblages from lakes located in the vicinity of the two main fjords (Woodfjorden and Wijdefjorden), analysis of similarities (ANOSIM) was run and similarity percentage analysis (SIMPER) was conducted to assess the level of compositional dissimilarity between and within the two groups. Differences in LOI, C_org_ and BSi concentrations among the three studied groups of samples were tested by analysis of variance (ANOVA) using Statistica version 5.5 software (StatSoft, Tulsa, OK, USA).

## Results

### Sediment and pore water characteristics

**Fig. 2:**
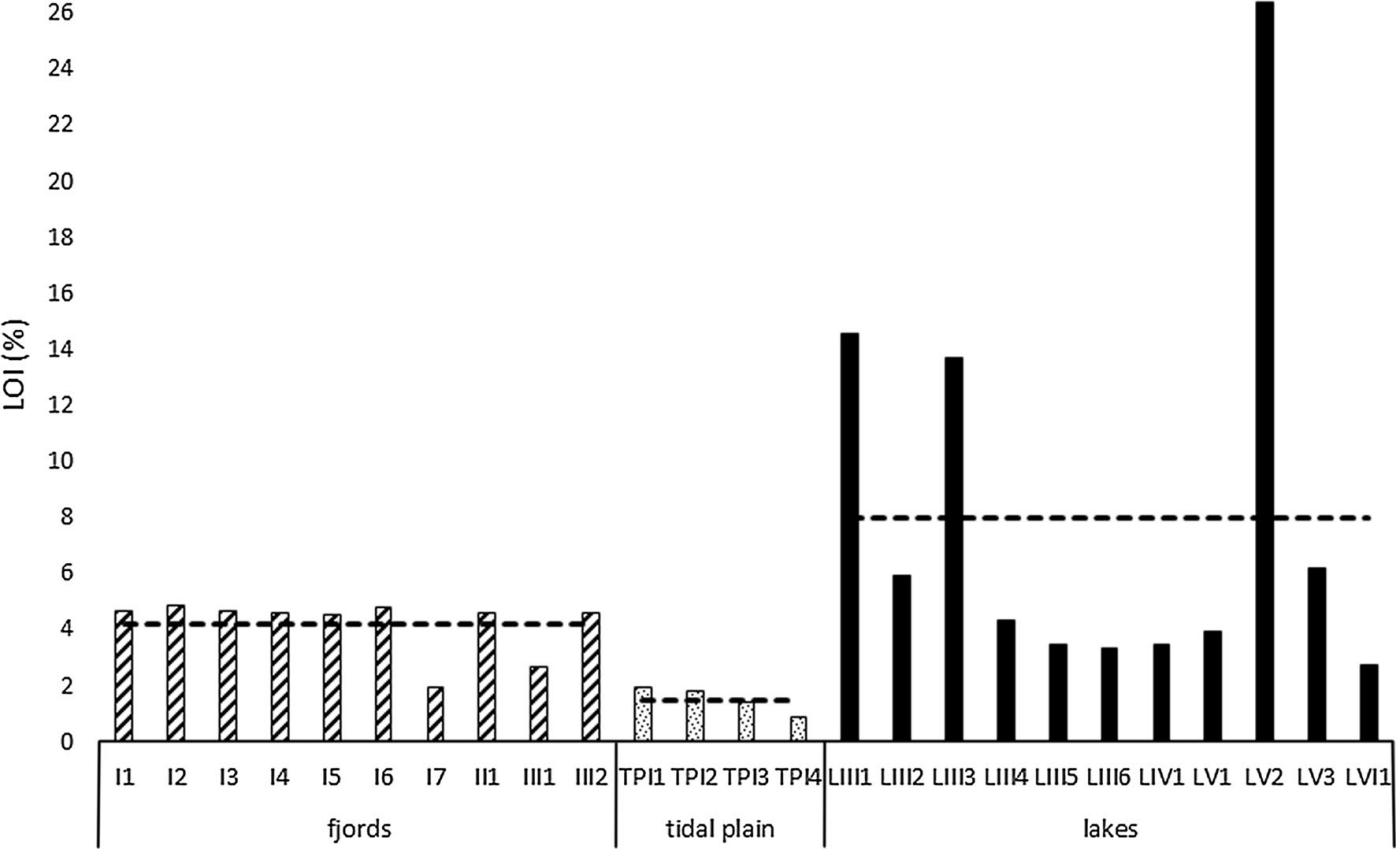
Loss on ignition (LOI) values for the sampling stations within the fjords, tidal plains and lakes in northern Spitsbergen in July 2005

**Fig. 3:**
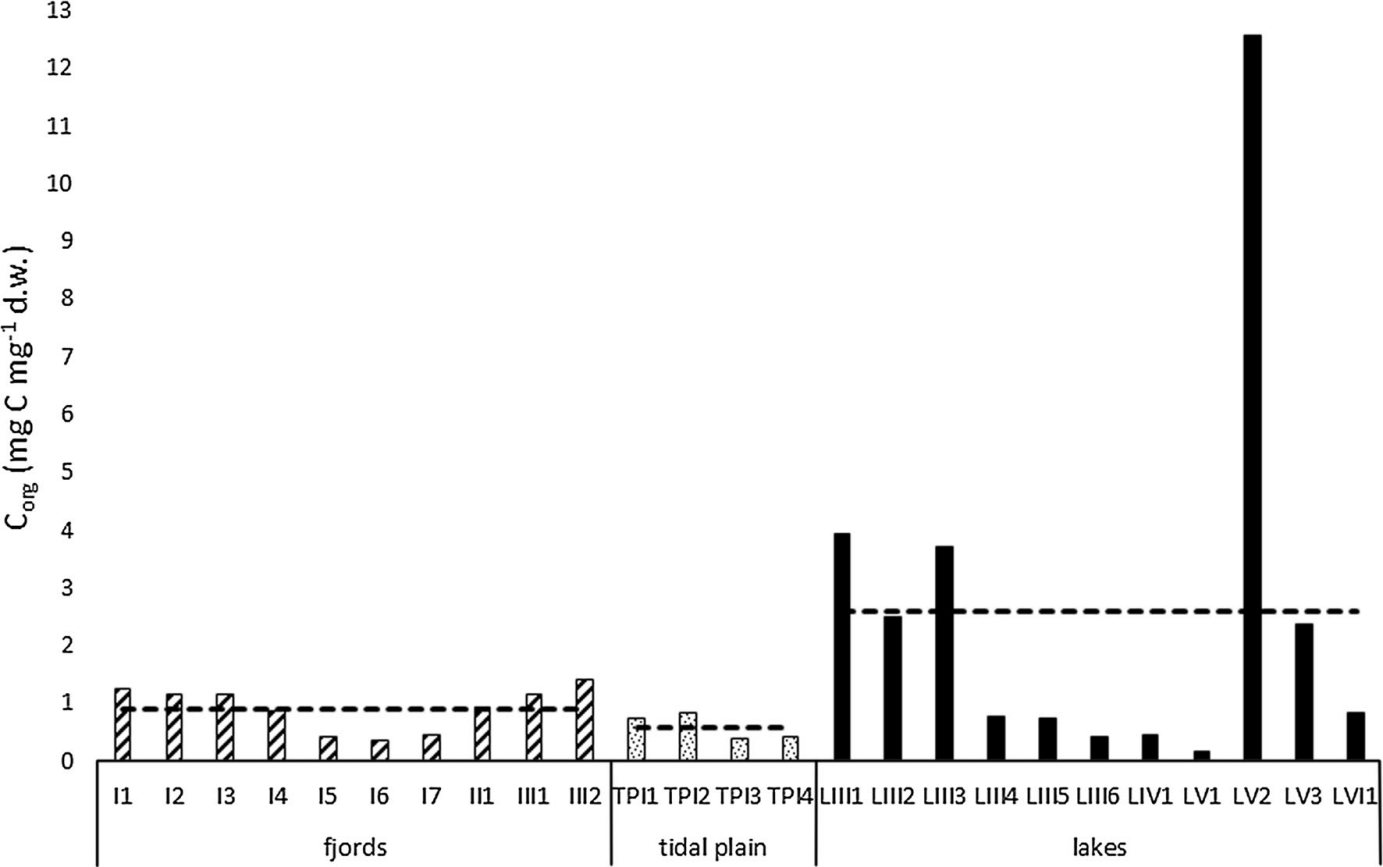
Organic carbon content in the surface sediments in fjords, tidal plains and lakes in northern Spitsbergen in July 2005

**Fig. 4:**
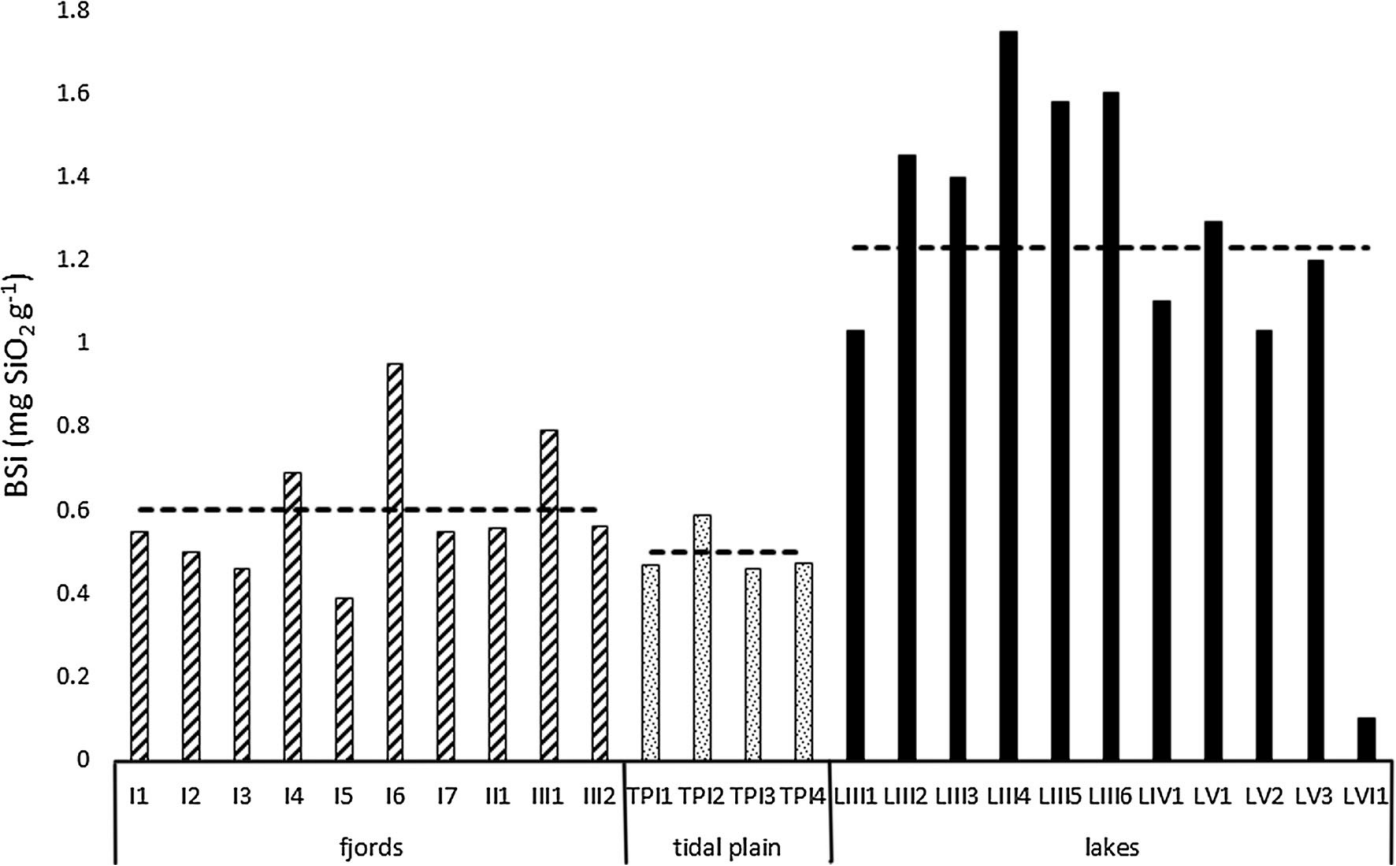
Biogenic silica content in the surface sediments in fjords, tidal plains and lakes in northern Spitsbergen in July 2005

**Fig. 5:**
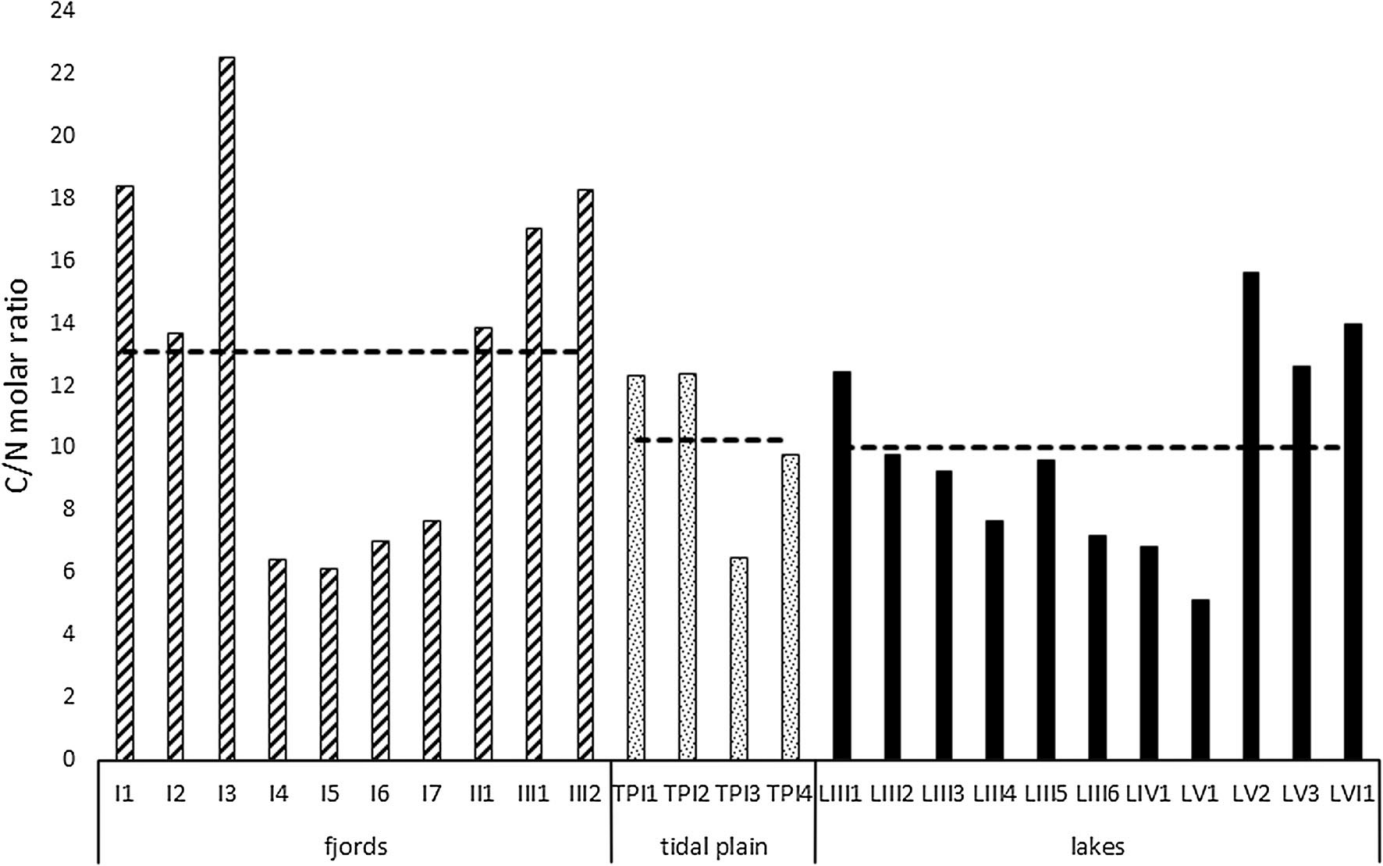
C:N molar ratio calculated for the surface sediments collected from fjords, tidal plains and lakes in northern Spitsbergen in July 2005

Sediment LOI values ranged from 1.95 to 4.9 % for fjords, from 0.9 to 1.95 % for tidal plains and from 2.78 to 26.41 % for lakes, with mean values of 4.2, 1.5 and 8 %, respectively (Fig. 2). Organic carbon content ranged from 0.36–1.41 mg C mg^−1^ d.w. for fjords, from 0.39–0.85 mg C mg^−1^ d.w. for tidal plains and from 0.18–12.57 mg C mg^−1^ d.w. for lakes. The highest mean value (2.6 mg C mg^−1^ d.w) characterized lake sediments, and the lowest (0.6 mg C mg^−1^ d.w.) was found in tidal plain sediments (Fig. 3). However, the observed differences in C_org_ concentration were not significant (ANOVA, *p* > 0.05). As shown in Fig. 4, the sample groups differed distinctly in terms of biogenic silica content. Although the lowest individual value of this parameter was observed in one of the lake samples (LVI1: 0.1 mg SiO_2_ g^−1^), overall BSi content was significantly higher (ANOVA, *p* < 0.001) in lake sediments (1.23 mg SiO_2_ g^−1^) than in both fjord and tidal plain sediments (0.6 and 0.5 mg SiO_2_ g^−1^, respectively). The values of the organic C/N molar ratios ranged from 5.1 (sample LV1) to 22.5 (sample I3; Fig. 5). The highest mean value (13.1) was obtained in samples collected in fjords and the lowest in those derived from lakes (10), but these differences were non-significant (ANOVA, *p* > 0.05). Concentrations of organic contaminants (PCBs, OCPs and PAHs) were generally very low, and it was assumed that they would not affect diatom communities (for detailed information on concentrations of organic contaminants, see Sapota et al. 2009).

### Diatom assemblages

**Fig. 6:**
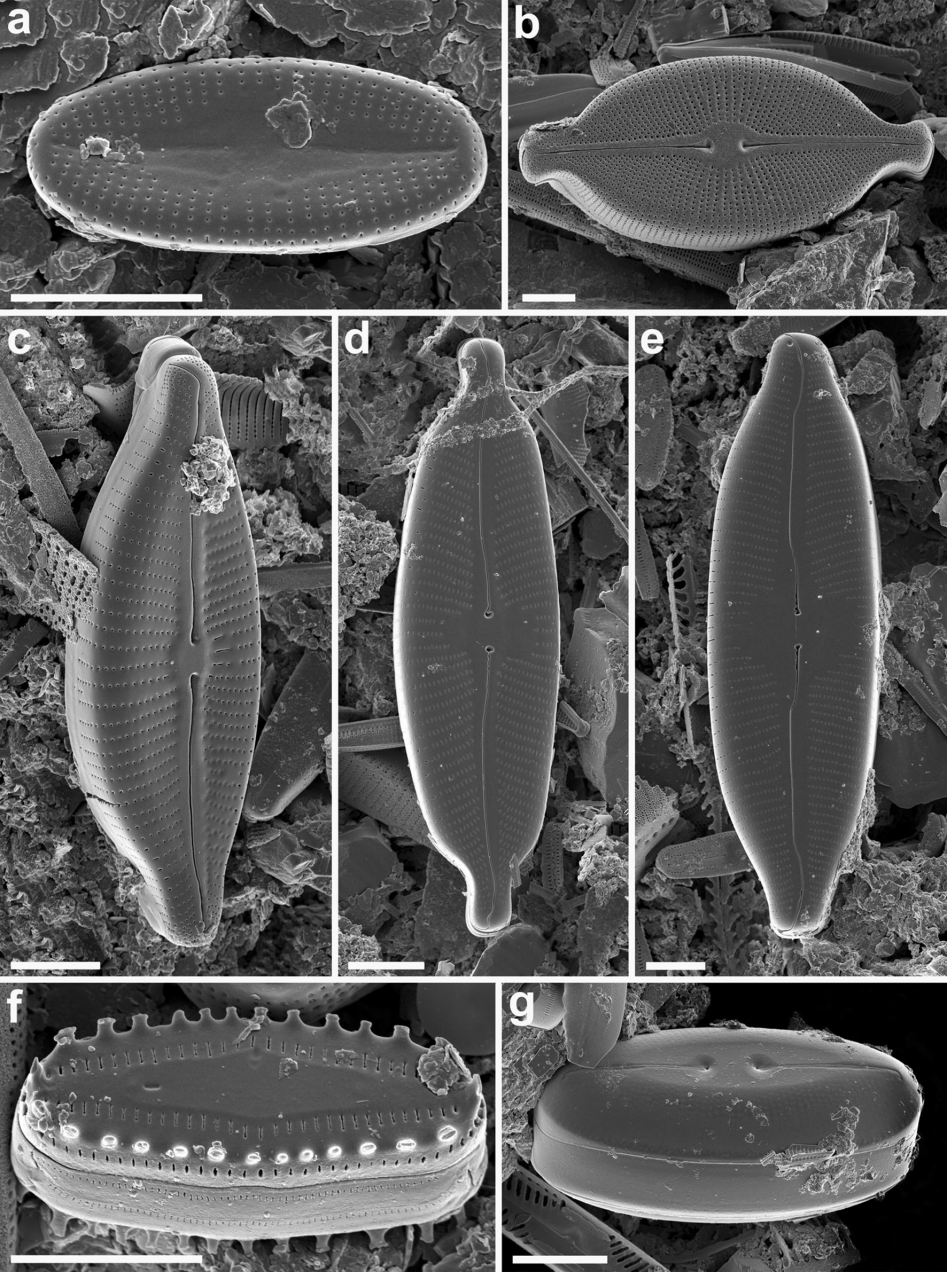
Freshwater diatoms from northern Spitsbergen (Svalbard). **a** *Psammothidium levanderi* (raphless valve), **b** *Cosmoneis pusilla*, **c** *Cymbella designata*, **d** *Cymbopleura naviculiformis*, **e** *C. tynnii*, **f** *Diadesmis gallica*, **g** *Diploneis ovalis* ssp. *arctica*. *Scale bars* = 5 µm

**Fig. 7:**
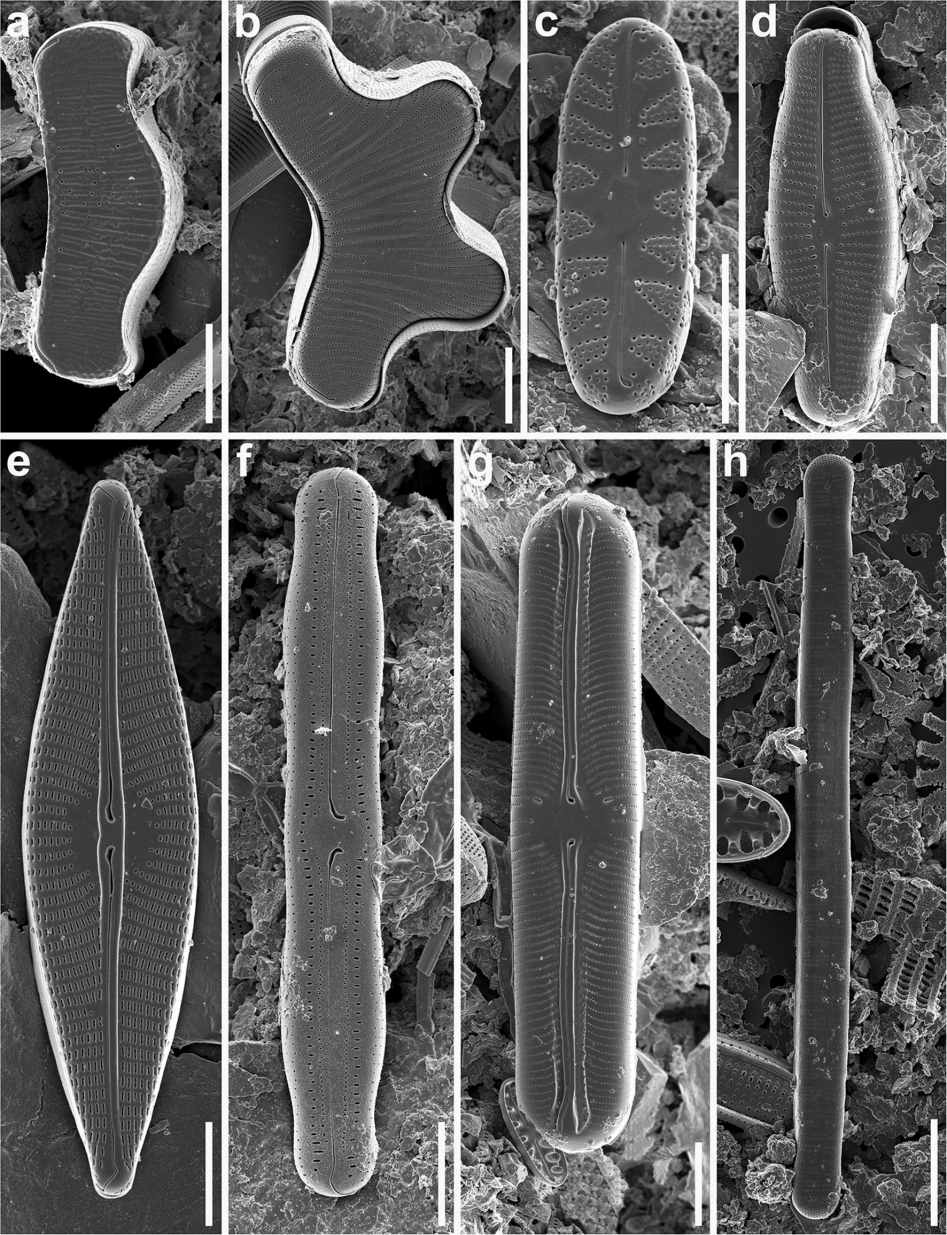
Freshwater diatoms from northern Spitsbergen (Svalbard). **a** *Eunotia grunowii*, **b** *E. papilio*, **c** *Hygropetra balfouriana*, **d** *Parlibellus protractoides*, **e** *Navicula trivialis*, **f** *Neidiopsis vekhovii*, **g** *Sellaphora laevissima*, **h** *Diatoma tenuis*. *Scale bars* = 5 µm

Samples taken from fjords and tidal plains were devoid of diatom valves or other siliceous remnants. A total of 96 diatom taxa belonging to 46 genera were identified in the lake samples (Table 3). Most diatom communities included a low number of taxa, with between two (lake LIV1) and 24 (lake LV3) taxa being identified in a single sample. Forty-four taxa had relative abundance greater than 1 % and only 11 over 5 % in any one sample. The large majority of diatom species recorded were truly benthic. Among the most important common taxa (taxa that occurred in more than 25 % of samples) were the most abundant *Staurosirella pinnata* (Ehrenberg) Williams & Round (with maximum relative abundance of 94.5; 44 % on average) and the most common *Achnanthidium minutissimum* (Kützing) Czarnecki (up to 75 % of total abundance; 14.2 % on average). *Nitzschia alpina* Hustedt (up to 60.6 %; 24.2 % on average), *Fragilaria capucina* Desmaziere (up to 8.9 %; 3.2 % on average) and *Encyonema minutum* (Hilse) D.G. Mann (up to 4 %; 1.1 % on average) were also frequently encountered. The most common taxa typical for the sites around the Woodfjorden coasts were *Cavinula pseudoscutiformis* (Hustedt) D.G. Mann & A.J. Stickle (up to 3.5 %; 1.9 % on average), *Encyonema reichardtii* (Krammer) D.G. Mann (up to 2.7 %; 1.7 % on average) and a group of unidentified *Navicula* spp. For the sites located around the Wijdefjorden coasts among the most important taxa were *Denticula kuetzingii* Grunow (up to 21.6 %; 9.3 % on average) and *Nitzschia inconspicua* Grunow (up to 8 %; 5.9 % on average). Five other taxa: *Encyonopsis microcephala* (Grunow) Krammer, *Eunotia incisa* W. Smith ex W. Gregory, *Humidophila contenta* (Grunow) Lowe, Kociolek, Johansen, Van de Vijver, Lange-Bertalot & Kopalová, *Hygropetra balfouriana* (Grunow ex Cleve) Krammer & Lange-Bertalot and *Karayevia oblongella* (Østrrup) Aboal occurred in more than 25 % of samples, but their relative abundances ranged between 0.9 % (*E. incisa* and *H. contenta*) and 2.7 % (*H. balfouriana*). Selected diatom taxa, rarely documented previously with SEM, are shown in Figs. 6 and 7.

**Table 3:** List of taxa with summary statistics: number of occurrences (*N*), maximum percentage value attained, lake with the maximum value and number of lakes where the taxon attained a value of 5 % or more

Taxa		Code	*N*	Max abundance		>5 %
				Value	Lake	
1.	*Achnanthes punctulata* Simonsen	Ach pun	1	20.7	III6	1
2.	*Achnanthidium helveticum* (Hustedt) Monnier, Lange-Bertalot & Ector	Ach hel	2	18.8	V1	2
3.	*A. minutissimum* (Kützing) Czarnecki	Ach min	11	75.0	V1	6
4.	*Alveovallum beyensii* Lange-Bertalot & Krammer	Alv bey	1	0.2	V3	0
5.	*Caloneis bacillum* (Grunow) Cleve	Cal bac	2	3.9	III4	0
6.	*C.* *silicula* (Ehrenberg) Cleve	Cal sil	1	3.5	III5	0
7.	*Cavinula cocconeiformis* (Gregory ex Greville) D.G.Mann & A.J.Stickle	Cav coc	1	0.2	III2	0
8.	*C. pseudoscutiformis* (Hustedt) D.G.Mann & A.J.Stickle	Cav pse	5	3.5	III3	0
9.	*Chamaepinnularia soehrensis* (Krasske) Lange-Bertalot & Krammer in Lange-Bertalot & Metzeltin	Cha soe	2	0.6	VI1	0
10.	*Cosmioneis pusilla* (W. Smith) D.G.Mann	Cos pus	1	0.4	V3	0
11.	*Craticula halophila* (Grunow) D.G.Mann	Cra hal	1	1.7	VI1	0
12.	*Cymbella* *designata* Krammer	Cym des	1	0.4	V2	0
13.	*Cymbopleura cuspidata* (Kützing) Krammer	Cym cus	2	0.2	V2	0
14.	*C. naviculiformis* (Auerswald ex Heiberg) Krammer	Cym nav	2	0.7	III5	0
15.	*C. tynnii* (Krammer) Krammer	Cym tyn	1	0.2	V3	0
16.	*Denticula kuetzingii* Grunow	Den kue	3	21.6	V2	2
17.	*Diadesmis gallica* W.Smith	Dia gal	1	0.2	V3	0
18.	*Diatoma* sp.	Dia sp	2	3.0	III6	0
19.	*D. tenuis* Agardh	Dia ten	2	0.9	V1	0
20.	*Diploneis ovalis* subsp. *arctica* Lange-Bertalot	Dip ova	1	0.2	V3	0
21.	*Encyonema minutum* (Hilse) D.G.Mann	Enc min	7	4.0	VI1	0
22.	*E. reichardtii* (Krammer) D.G.Mann	Enc rei	3	2.7	III1	0
23.	*E. silesiacum* (Bleisch) D.G.Mann	Enc sil	1	1.0	III3	0
24.	*Encyonopsis microcephala* (Grunow) Krammer	Enc mic	3	1.8	III5	0
25.	*Eucocconeis flexella* (Kützing) Meister	Euc fle	2	0.6	VI1	0
26.	*E. laevis* (Øestrup) Lange-Bertalot	Euc lae	2	3.4	VI1	0
27.	*Eunotia grunowii* Å.Berg	Eun gru	1	0.2	V3	0
28.	*E. incisa* W.Smith ex W.Gregory	Eun inc	3	0.9	V2	0
29.	*E. papilio* (Ehrenberg) Grunow	Eun pap	1	0.2	V3	0
30.	*E. praerupta* Ehrenberg	Eun pra	2	0.9	V1	0
31.	*Fragilaria capucina* Desmazières	Fra cap	4	8.9	III3	1
32.	*F. capucina* var. *rumpens* (Kützing) Lange-Bertalot	Fra cvr	1	0.2	III1	0
33.	*Gomphonema* cf. *angustum* Agardh	Gom can	2	0.7	III5	0
34.	*G.* cf. *arctica* Grunow	Gom car	2	0.2	III5	0
35.	*G. truncatum* Ehrenberg	Gom tru	1	0.6	VI1	0
36.	*Handmannia antiqua* (W.Smith) Kociolek & Khursevich	Han ant	1	0.4	V2	0
37.	*Hannaea arcus* (Ehrenberg) R.M.Patrick	Han arc	1	0.2	VI1	0
38.	*Hantzschia* sp.	Han sp	1	0.2	V3	0
39.	*Humidophila brekkaensis* (Petersen) Lowe, Kociolek, Johansen, Van de Vijver, Lange-Bertalot & Kopalová	Hum bre	1	1.6	V3	0
40.	*H. contenta* (Grunow) Lowe, Kociolek, Johansen, Van de Vijver, Lange-Bertalot & Kopalová	Hum con	3	0.9	V1	0
41.	*H. perpusilla* (Grunow) Lowe, Kociolek, Johansen, Van de Vijver, Lange-Bertalot & Kopalová	Hum per	1	0.2	V3	0
42.	*Hygropetra balfouriana* (Grunow ex Cleve) Krammer & Lange-Bertalot	Hyg bal	5	2.7	V3	0
43.	*Karayevia clevei* (Grunow) Round & Bukhtiyarova	Kar cle	2	1.1	VI1	0
44.	*K.* *oblongella* (Østrrup) Aboal	Kar obl	3	1.4	III3	0
45.	*Meridion circulare* (Greville) C.Agardh	Mer cir	1	0.2	VI1	0
46.	*Microcostatus krasskei* (Hustedt) J.R.Johansen & J.C.Sray	Mic kra	1	1.6	V3	0
47.	*Muelleria bachmannii* (Hustedt) S.A.Spaulding & E.F.Stoermer	Mue bac	1	0.2	V3	0
48.	*Navicula cari* Ehrenberg	Nav car	2	0.2	III5	0
49.	*N.* cf. *bremensis* Hustedt	Nav cbr	2	2.0	III6	0
50.	*N.* cf. *cryptocephala* Kützing	Nav ccr	1	0.4	III2	0
51.	*N.* cf. *tenerrima* Hustedt	Nav cte	2	0.6	VI1	0
52.	*N. cryptocephala* Kützing	Nav cry	1	0.6	III2	0
53.	*N. digituloides* Lange-Bertalot	Nav dig	1	2.7	III1	0
54.	*N. halophiloides* Hustedt	Nav hal	1	1.3	V2	0
55.	*N. phyllepta* Kützing	Nav phy	1	1.6	V3	0
56.	*N. radiosa* Kützing	Nav rad	1	0.2	V2	0
57.	*N.* *trivialis* Lange-Bertalot	Nav tri	1	0.2	III1	0
58.	*Navicula* sp. 1	Nav sp1	4	4.0	III2	0
59.	*Navicula* sp. 2	Nav sp2	3	4.7	III3	0
60.	*Navicula* sp. 3	Nav sp3	4	0.5	III6	0
61.	*Neidium ampliatum* (Ehrenberg) Krammer	Nei amp	1	0.2	III2	0
62.	*N. perminutum* Cleve-Euler	Nei per	1	1.9	V3	0
63.	*Neidiopsis vekhovii* (Lange-Bertalot & S.I.Genkal) Lange-Bertalot	Nei vek	1	0.2	V3	0
64.	*Nitzschia alpina* Hustedt	Nit alp	8	60.6	III6	6
65.	*N. amphibia* Grunow	Nit amp	1	0.2	III2	0
66.	*N. inconspicua* Grunow	Nit inc	3	8.5	VI1	2
67.	*N. linearis* (Agardh) W.Smith	Nit lin	2	0.6	VI1	0
68.	*N. palea* (Kützing) W.Smith	Nit pal	1	0.4	V3	0
69.	*Parlibellus protractoides* (Hustedt) Witkowski & Lange-Bertalot	Par pro	1	0.2	VI1	0
70.	*Pinnularia divergentissima* (Grunow) Cleve	Pin div	1	0.5	III6	0
71.	*P. microstauron* (Ehrenberg) Cleve	Pin mic	2	7.0	V3	1
72.	*P. obscura* Krasske	Pin obs	2	0.5	III6	0
73.	*P.* *streptoraphe* Cleve	Pin str	2	0.4	III3	0
74.	*Placoneis clementis* (Grunow) E.J.Cox	Pla cle	2	1.0	III6	0
75.	*P. placentula* (Ehrenberg) Mereschkowsky	Pla pla	1	0.4	III3	0
76.	*Planothidium conspicuum* (Mayer) Aboal	Pla con	1	7.4	V3	1
77.	*P. delicatulum* (Kützing) Round & Bukhtiyarova	Pla del	1	0.2	VI1	0
78.	*P. holstii* (Cleve) Lange-Bertalot	Pla hol	1	0.8	V3	0
79.	*P. lanceolatum* (Brébisson ex Kützing) Lange-Bertalot	Pla lan	1	0.4	V3	0
80.	*P. pericavum* (J.R.Carter) Lange-Bertalot	Pla per	1	1.0	III6	0
81.	*Psammothidium chlidanos* (Hohn & Hellerman) Lange-Bertalot	Psa chl	2	5.4	V3	1
82.	*P. frigidum* (Hustedt) Bukhtiyarova & Round	Psa fri	1	0.2	VI1	0
83.	*P.* *levanderi* Hustedt	Ach lev	2	0.5	III6	0
84.	*P. subatomoides* (Hustedt) Bukhtiyarova & Round	Psa sub	2	1.3	III1	0
85.	*Pseudostaurosira brevistriata* (Grunow) D.M.Williams & Round	Pse bre	2	2.3	VI1	0
86.	*Reimeria* *capitata* (Cleve-Euler) Levkov & Ector	Rei cap	1	1.7	VI1	0
87.	*R. sinuata* (Gregory) Kociolek & Stoermer	Rei sin	2	4.0	III1	0
88.	*Rossithidium petersenii* (Hustedt) Round & Bukhtiyarova	Ros pet	2	1.6	III5	0
89.	*Sellaphora laevissima* (Kützing) D.G.Mann	Sel lae	2	3.9	III3	0
90.	*S. radiosa* (Hustedt) H.Kobayasi	Sel rad	2	0.5	III6	0
91.	*Stauroneis alpina* Hustedt	Sta alp	1	2.1	V3	0
92.	*S. anceps* Ehrenberg	Sta anc	1	0.4	V2	0
93.	*Staurosira construens* Ehrenberg	Sta con	1	1.1	III1	0
94.	*S. construens* var. *venter* (Ehrenberg) P.B.Hamilton	Sta vvc	1	3.0	III6	0
95.	*Staurosirella pinnata* (Ehrenberg) Williams et Round	Sta pin	10	94.5	V1	8
96.	*Tabellaria flocculosa* (Roth) Kützing	Tab flo	3	4.1	III3	0

**Fig. 8:**
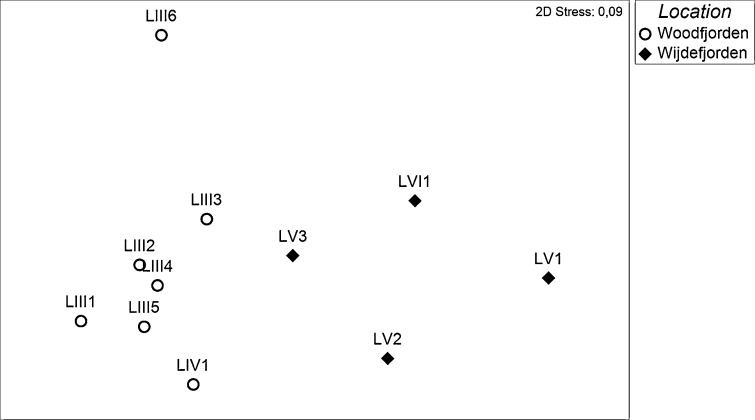
NMDS scaling plot based on Bray-Curtis similarities of the diatom data consisting of the presence-absence of diatoms in lacustrine samples

The nMDS plot revealed differences among sample groups (Fig. 8). The optimal 2-D solution was found by minimizing the stress value (2D stress = 0.09). Sites located along the Woodfjorden coast formed a distinct group (LIII and LIV) from those situated in the vicinity of Wijdefjorden (LV and LVI). ANOSIM results indicated significantly greater differences in the diatom assemblages between these groups than within (*p* < 0.05) and confirmed the significance of the observed pattern (global *R* = 0.656). According to SIMPER, the average similarity values within the sample groups were 47.4 and 37.1 % for the Woodfjorden and Wijdefjorden group, respectively, and the average dissimilarity between the groups was 76.8 %.

**Fig. 9:**
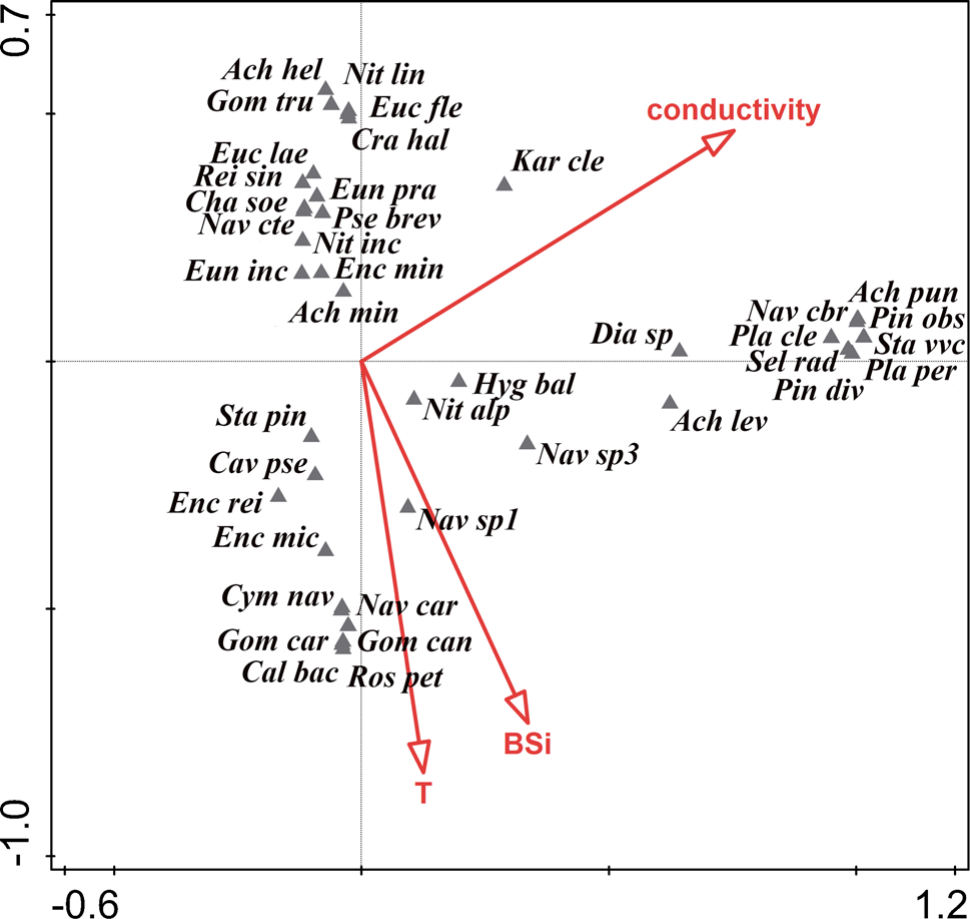
*Biplot diagram* from CCA summarizing the variation in diatom species composition explained by environmental variables. Arrows represent environmental variables indicated as significant predictors of diatom composition. Score scaling is focused on diatom scores (standardized). Only the 40 best fitting taxa are shown. Eigenvalues: 0.5509, 0.4429, 0.2715

Only three of the measured variables were identified by the interactive forward selection procedure as significant predictors of diatom composition. These were water conductivity (*p* < 0.01; explaining 14.9 % of the observed variation), biogenic silica concentration (*p* < 0.05; explaining 14.5 % of the observed variation) and water temperature (*p* < 0.05; explaining 11 % of the observed variation; Fig. 9).

## Discussion

### Diatoms in lakes

Ninety-six diatom taxa belonging to 46 genera were found in samples collected from 11 lakes located in northern Spitsbergen. Three dominant species, *Achnanthidium minutissimum*, *Staurosirella pinnata* and *Nitzschia alpina*, occurred in at least eight of the lakes investigated. These species are known to have a wide geographical distribution, being especially abundant in other cold or high altitude regions of Europe, Asia and America (e.g. Krammer and Lange-Bertalot 1991b; Paull et al. 2008; Robinson et al. 2010). They are well adapted to low nutrient and light conditions and are common in sites with low human impact (Van Dam et al. 1994; Johnson et al. 2009). On the other hand, *A. minutissimum* sensu stricto is often recognized as an indicator of disturbed, unstable habitats (Biggs et al. 1999; Wang et al. 2009; Mihaljević and Pfeiffer 2012). In this study, diatom counts were made using light microscopy and it is probable that, due to a very small cell size, different *Achnanthidium* species could not be distinguished. Recently, a new *Achnanthidium* species was described from Spitsbergen (Pinseel et al. 2015) and it is possible that *Achnanthidium minutissimum* reported here was in fact a cluster of several *Achnanthidium* taxa.

The lack of planktonic diatoms might be indicative of a high level of disturbance, preventing planktonic species from reaching their carrying capacity or making their growth impossible (Sommer 1993; Reynolds 1994). The relatively small number of taxa comprising assemblages in these lakes (from 2 to 23, average 16) and dominance of diatoms from the low-profile guild (after Passy 2007), e.g. from the genera *Achnanthes*, *Achnanthidium*, *Reimeria*, also indicate conditions of high disturbance, which appropriately adapted species can resist.

Jones and Birks (2004), who examined surface sediments from 23 lakes in western Spitsbergen, found 182 diatom taxa, with benthic species (belonging mainly to *Fragilaria*, *Navicula* and *Achnanthes* genera) dominating all sites. Samples from only three sites contained planktonic species belonging to *Cyclotella* genus. The higher number of diatom taxa found by the authors is undoubtedly related to the higher number of lakes sampled and much larger (and more diverse) sampling area considered in their study. In addition, many of the sites examined by Jones and Birks (2004) were located relatively close to human settlements, where diatom species diversity might be higher due to introductions of non-indigenous taxa through anthropogenic means. Only 40 % of taxa found in our study were also observed by Jones and Birks (2004), which may indicate that Svalbard lacustrine communities differ locally.

Recently, Pinseel et al. (2016) reported especially high diatom diversity in samples collected from Petuniabukta (Billefjorden, central west Spitsbergen). In 87 samples of various substrates (epipelon, epilithon and epiphyton) collected from 53 lakes and ponds, they found 310 diatom taxa belonging to 59 genera. More than one-third of the taxa observed could not be satisfactorily assigned to any known species. The study included detailed SEM analyses of diatom frustule ultrastructure and applied extended counting procedure, where entire slides were additionally scanned for rare taxa. This, as well as the fact that all sampling sites chosen by Pinseel et al. (2016) were located in an area that has undoubtedly been influenced by human activities for the last 100+ years, might have contributed to the high number of taxa and morphospecies discovered. The ongoing upheavals in diatom taxonomy continuously shift taxonomic concepts, while it is further likely that new analytical tools (including molecular methods) will profoundly impact our perception of global diatom diversity and their dispersal strategies.

### Diatoms and environmental variables

Although situated within the same geographical area (northern Spitsbergen) and thus subjected to the similar general climatic conditions, the studied lakes differed in their limnological features, with these differences likely to affect diatom growth conditions (Sommer 1993; Sommaruga 2015; Pinseel et al. 2016; Pla-Rabés et al. 2016). Statistical analyses performed (nMDS, ANOSIM, SIMPER) indicated that the diatom assemblages found in lakes located in the vicinity of the two main fjords clearly differed. Assemblages from the Woodfjorden region were generally characterized by the presence of *C. pseudoscutiformis* and *E. reichardtii*, accompanied by *Navicula* spp., associated with relatively low conductivity (34–58.7 µS cm^−1^) and near-neutral pH (7.2–7.5). Assemblages found in the Wijdefjorden area had different taxonomic composition, typically with *D. kuetzingii* and *N. inconspicua* in the three lakes with relatively high water conductivity (>184 µS cm^−1^) and slightly alkaline pH (7.5–8.1). Further, the relatively low values of similarity within the two groups (47.35 and 37.14 % for Woodfjorden and Wijdefjorden, respectively) also indicate a certain level of distinctness of the diatom assemblages in each of the lakes studied.

Among the environmental factors considered in this study, three—water conductivity, biogenic silica concentration and water temperature—were identified as significant predictors of the diatom community species composition and structure. Numerous studies have confirmed the importance of these and other environmental variables for diatom assemblage development (e.g. Picińska-Fałtynowicz 1988; Bouchard et al. 2004; Jones and Birks 2004; McKay et al. 2008; Majewska et al. 2012). It should be noted, however, that due to the small sample size (*n* = 11), detection of any less pronounced effects was unlikely.

The BSi concentration in pore water is a direct result of diatom (and/or other siliceous organisms) skeleton decomposition; thus, it should be expected to be positively correlated with diatom abundance (Conley and Schelske 2001). Nevertheless, different diatom taxa exhibit different levels of frustule silicification, and differences in pore water BSi concentration may underlie general differences in diatom community composition and structure (Ryves et al. 2001). On the other hand, dissolution rates of silica walls of diatoms depend on pore water chemistry (its conductivity and pH) and temperature, and thus the relatively high predictive potential of BSi concentration should be interpreted with caution (Lewin 1961; Coradin and Lopez 2003). Both water temperature and conductivity variations could be directly impacted by global climate changes, which suggests that Arctic diatom communities will be strongly subjected to those changes and, at the same time, will reflect the new environmental conditions. This has already been observed by Douglas et al. (1994) and Gajewski et al. (1997), who used paleolimnological data to assess the recent environmental changes in the Canadian High Arctic, concluding that differences in diatom community composition found in sediment cores indicated clearly significant changes in habitat conditions beginning in the nineteenth Century.

The organic matter concentration (estimated as LOI) was generally higher in lake sediments than in the other sediment types examined. This was probably due to both autochthonous organic matter production and the inflow of allochthonous organic matter, which enters lakes during the spring snowmelt. Although melting snow does not contain large amounts of organic matter, meltwater can subsequently carry organic particles from soil and terrestrial habitats into the lakes (Everett et al. 1996; Oswood et al. 1996; Hadley et al. 2013). The differences in LOI observed among lake samples are likely to be underlain by their different morphology and hydrological conditions (Sapota et al. 2009) and by the different thaw depth and routes that meltwater takes to reach them (Oswood et al. 1996; Michaelson et al. 1998).

### Diatom absence in tidal plain and fjord sediments

Sediments collected from tidal plains and fjords proved to be devoid of siliceous remnants, even though the amount of sediment taken for diatom analysis (ca. 150 g) is considered high enough to ensure the sample representativeness. BSi may be a good proxy for diatom abundance (Conley and Schelske 2001), and its low concentrations found in pore waters from tidal plains and fjords lend further support to our observations. Similarly, low BSi values have been reported from other Arctic marine regions, such as the Laptev Sea (Heiskanen and Keck 1996). Surface sediments in Arctic tidal plains can be unstable and turbid habitats due to debris and sediment transportation with melting water from glaciers, redeposition of loose sediment during floods or settling of fine particles in the course of flocculation when freshwater mixes with marine water in the fjords (Hop et al. 2002; Svendsen et al. 2002; Zajączkowski 2008). Although many diatom species are considered as highly adapted to high disturbance and rapidly changing environments (Margalef 1978), constant grounding and scouring by ice-rafted debris and sediments along with limited nutrient resources can prevent them from settlement and curtail development of initial assemblages. Microphytobenthic biofilms in tidal plains have been investigated extensively worldwide, but most of these studies focus on cold-temperate and tropical waters (e.g. Haubois et al. 2005; Karsten et al. 2012). Low values of LOI and C content in tidal plain sediments obtained in this study may be an indicator of poor development of local micro- and macrocommunities. Indeed, previous investigations in Arctic tidal plains have shown that the number of meio- and macrofauna can be extremely low in comparison with tidal plains in other regions (Jan Węsławski personal communication, Wojtasik unpubl.). Exposure of the tidal plain to wave action may also be important and, in heavy wave action systems worldwide, the absence of mud and mobile fauna is observed (McLachlan 1983).

Sampling sites in fjords were located mainly in deeper waters (below 40 m) where light deficiency significantly limits development of benthic photosynthetic communities (Glud et al. 2002; Hegseth and Sundfjord 2008). Nonetheless, the lack of any planktonic or epiphytic diatom remnants was unexpected.

It has been shown that the concentration of biogenic silica in sediments is strictly correlated with the amount of preserved diatom frustules (Conley 1998; Lin et al. 2001). The slightly higher C/N molar ratio and significantly lower biogenic silica concentrations found in the tidal plain and fjord sediments in comparison with lake sediments may indicate a different source of organic matter other than diatoms influencing the tidal plains and fjords. This may suggest that in the marine and coastal sites investigated here diatoms are not a dominant group of primary producers, as has also been observed in other Svalbard fjords (Hasle and Heimdal 1998; Wängberg et al. 2008; Iversen and Seuthe 2011). Due to the constantly high sun angle in summer as well as the significant reduction in stratospheric ozone, aquatic habitats located at high northern latitudes are characterized by extreme light conditions, which may be especially unfavourable for typically planktonic diatom growth (Wängberg et al. 2008). Several studies indicate that high levels of UV-B radiation observed at high latitudes induce important changes in the composition of the local phytoplankton communities, and in particular that large-celled diatoms are replaced by better adapted small flagellate algae (Mostajir et al. 1999; Mousseau et al. 2000; Davidson and Belbin 2002).

Winnowing, tidal and storm-generated currents, glacial processes or floating ice may also significantly affect the accumulation rates and limit suspended particles from being deposited onto the underlying seafloor (Campbell 1979; La Fon 1981; Anderson et al. 1984; Stein 2008). It is therefore probable that the sheer amount of alluvial/glacial outwash entering coastal waters dwarfed any biogenic input, making the grab sample size too small to contain any deposited diatoms.

Several studies have emphasized that, globally, biogenic silica preservation efficiency is highly variable and depends on many, often unidentified, factors (e.g. Nelson 1988; DeMaster et al. 1996; DeMaster 2002; Loucaides et al. 2008). Among the important factors that undoubtedly affect BSi deposition and preservation are temperature, water chemistry (BSi dissolution rates are far higher in sea water than in freshwater; Loucaides et al. 2008), species composition of the local siliceous assemblages and the type of their vertical transport in the water column, which may involve sinking of discrete particles, aggregates or faecal pellets (DeMaster et al. 1996). In the world ocean, which is generally under-saturated with respect to BSi, the dissolution of silica debris takes place over different timescales, and about 50 % of BSi produced in the euphotic zone is dissolved before reaching the seabed (Treguer et al. 1995). In the seabed, BSi dissolution rates can be higher, even reaching 100 % (DeMaster et al. 1996). The higher pH of sea water in synergy with high concentration of Na^+^ and Mg^2+^ cations that catalyse the hydrolysis of siloxane bonds present in diatom shells enhances significantly the diatom frustule dissolution (Loucaides et al. 2008). Many marine planktonic species are lightly silicified, and their frustules are decomposed in the water column before reaching the seabed (Kamatani 1982). This process is enhanced by intense grazing, which characterizes the shallow-water coastal zone (Treguer et al. 1995; DeMaster et al. 1996; Van Cappellen et al. 2002).

## Conclusions

Diatom communities from the northern Spitsbergen lakes studied had limited species diversity, but were spatially distinct and dominated by natural disturbance-adapted taxa. Among the environmental variables tested, water conductivity, temperature and BSi concentration were indicated as significant predictors of diatom community composition. This suggests that these freshwater communities may be useful as bioindicators of past and future climate change. The lack of diatom remnants and relatively low BSi concentrations in tidal plain and fjord sediments and pore waters may suggest that diatoms are not the most important group of photosynthetic microalgae in these areas and/or that the lightly silicified and potentially heavily grazed marine diatom frustules decompose before reaching the seabed.
